# Bio-Prospecting of a Few Brown Seaweeds for Their Cytotoxic and Antioxidant Activities

**DOI:** 10.1093/ecam/neq024

**Published:** 2011-03-10

**Authors:** Rashmi C. Vinayak, A. S. Sabu, Anil Chatterji

**Affiliations:** Biological Oceanography Division, National Institute of Oceanography, Dona Paula, Goa 403004, India

## Abstract

Methanolic extracts (MEs) of seven brown seaweeds occurring in the Indian coastal waters were screened for their cytotoxic and antioxidant properties following various assays. The methanolic extracts of seaweeds in the order of *Dictyopteris australis > Spatoglossum variabile > Stoechospermum marginatum > Spatoglossum aspermum* showed significant cytotoxic activity. A very high DPPH radical scavenging activity was exhibited by the methanolic extracts prepared from *St. marginatum, Padina tetrastromatica, Dictyopteris delicatula* and *S. aspermum*. The total phenolic content of the MEs varied from 13.19 ± 0.32 to 25.29 ± 0.445 gallic acid equivalents (mg g^−1^ of methanolic extract). The reducing power assay indicated a dose dependency, at concentrations of 0.1, 0.5 and 1.0 and 2.0 mg mL^−1^ of MEs and decreased in the following order: *Butylated hydroxy toluene > P. tetrastromatica > D. delicatula > S. aspermum > S. variabile > S. marginatum > D. australis > S. marginatum*. Furthermore, *D. australis*, *S. aspermum, S. variabile* and *S. marginatum* demonstrated good metal ion chelating properties. All the above evidences suggest that, the antioxidant compounds found in brown seaweeds scavenge free radicals through effective intervention. This decisively promotes them as a potential source of natural antioxidants.

## 1.Introduction

Reactive oxygen species (ROS) is a collective term used for radicals, for example, superoxide radical, hydroxyl radical, peroxyl radical or reactive non-radical compounds such as singlet oxygen, peroxynitrite or hydrogen peroxide; generally produced by endogenous and exogenous factors. These ROS are highly reactive, neutral, short lived and unstable oxygen containing molecules with an inherent capacity to form a final stable configuration. All such ROS possess the ability to cause far-reaching oxidative damage to healthy cells by reacting with their nucleic acids, proteins, lipids, enzymes and other small cellular molecules. They have been implicated in the etiology of several degenerative disease conditions, including cancer, cardiovascular diseases, rheumatoid arthritis, cataracts, immune system decline, liver diseases, diabetes mellitus, renal failure, brain dysfunction and aging [1]. Moreover, ROS-mediated oxidations are also responsible for the rancidity of unpreserved foods rich in unsaturated fatty acids. Synthetic antioxidants such as propyl gallate, butylated hydroxyanisol (BHA), butylated hydroxytoluene (BHT) and *tert*-butyl hydroquinone (TBHQ) are commonly used to control lipid oxidation in foods but are suspected to be responsible for liver damage and carcinogenesis [2, 3]. All these concerns regarding the synthetic antioxidants, together with consumers' preference for natural food ingredients, have reinforced the current attention toward the development of alternative natural antioxidants.

Over the past several decades, seaweeds and their extracts have generated an enormous amount of interest in the pharmaceutical industry as a fresh source of bioactive compounds with immense medicinal potential [4]. Seaweeds are rich in antioxidants such as carotenoids, pigments, polyphenols, enzymes and diverse functional polysaccharides [5–9]. This has been evidenced by recent investigations reporting a multitude of antioxidant compounds; for example, phylopheophytin in *Eisenia bicyclis* [10], phlorotannins in *Sargassum kjellamanianum* [11], fucoxanthin in *Hijikia fusiformis* [12], a low-molecular-weight sulfated polysaccharide from *Laminaria japonica* [13] and mycosporin-like amino acids (MAAs) from red seaweeds [14]. Their activities have been reported through a range of mechanisms, such as prevention of chain initiation, decomposition of peroxides, prevention of continual hydrogen abstraction, free radical scavenging, reducing power and binding of transition metal ion catalysts [15, 16]. As a result, a lot of attention has centered on seaweeds as alternative resources for extracting natural antioxidants.

In India seaweeds are mainly exploited as a source of phycocolloids such as agar-agar, alginate and carrageenan and not for their beneficial aspect with respect to food and medicine [17]. Further information on the bioutilization of Indian seaweeds is limited as not much has been done to systemically study their therapeutic potential [18–21].

The present study was undertaken to investigate cytotoxic activity of seven brown seaweed methanolic extracts (MEs) by Brine shrimp lethality assay and antioxidant properties by 1,1-diphenyl-2-picrylhydrazyl (DPPH) radical scavenging assay, reducing power assay and metal chelation assay *in vitro*. The total content of phenolic compounds in the extract was also determined. The results are hoped to provide an insight into the bioactive potential of Indian seaweed extracts.

## 2. Methods

### 2.1. Collection of Seaweeds

Seven species of seaweeds were collected from the coasts of Goa and Maharashtra during the low tide and then transported immediately to the Aquaculture laboratory of National Institute of Oceanography (NIO), Goa, where they were identified (Table 1). The samples were washed thoroughly with freshwater to remove salt, sand and epiphytes, dried at room temperature and stored at −20°C until further use.

### 2.2. Preparation of Extract

Dried and powdered seaweed samples (20 g) were suspended in 500 ml methanol at room temperature for 24 h extraction. The extraction was repeated twice and the total organic extracts (1.5 L) obtained were pooled, filtered and evaporated to dryness under pressure using a rotary evaporator (Roteva, India) to get a semi-solid residue. The product thus obtained was designated as the ME and stored at –20°C until further analysis.

#### 2.2.1. In Vitro Cytotoxicity Assay

Brine Shrimp Lethality Test. The toxicity against *Artemia salina* nauplii (Brine shrimp) was tested according to the method of Sam et al. [22] with minor modifications. Dried cysts were hatched (1 g cyst per liter) in sterile filtered seawater (0.22 *μ*m) at 27–30°C with strong aeration, under a continuous light regime. Approximately 12 h after hatching, the phototrophic nauplii were collected with a pipette and concentrated in a small vial. Each test consisted of exposing groups of 20 nauplii to various concentrations (50, 100, and 500 *μ*g) of the ME of individual seaweeds. The toxicity was determined after 6, 18, and 24 h of exposure by counting the number of survivors and calculating the percentage of mortality. Potassium dichromate (K_2_Cr_2_O_7_) and Milli-Q water were used as a positive and negative control, respectively. Larvae were considered dead if they did not exhibit any internal or external movement during the observation. Mortality below 50% was considered non-cytotoxic; mortality higher than 50% but below 75% was considered mildly cytotoxic; while mortality higher than 75% was considered as highly cytotoxic.

#### 2.2.2. In Vitro Antioxidant Assays

Total Phenolic Content. The total phenolic content (TPC) was determined by the Folin-Ciocalteu method as described by Sellappan and Akoh [23]. Seaweed extracts (0.5 mL) or gallic acid standard solution were mixed with 2.5 mL of Folin-Ciocalteu's reagent (FCR, 1:10 dilution) and left to stand for 8 min at room temperature to facilitate the FCR to react with the oxidizable substances or phenolates. Then, 2.0 mL of Na_2_CO_3_ (7.5% solution in water) was added to neutralize the residual reagent. After incubating for 2 h at room temperature, the absorbance was measured at 760 nm. Results were expressed as mg Gallic acid equivalents (GAE) per gram of seaweed extract.

#### 2.2.3. DPPH Radical Scavenging Assay

The scavenging effects of samples for 1,1-diphenyl-2-picrylhydrazyl hydrate (DPPH*) was determined spectrophotometrically according to the method of Duan et al. [24]. A 2 mL aliquot of test sample (in methanol) was added to 2 mL of 0.16 mM DPPH methanolic solution. The mixture was vortexed for 1 min and then left to stand at room temperature for 30 min in the dark. The absorbance was read at 517 nm and percentage of radical scavenging effect was calculated using the following equation:

(1) $$\text{Scavenging}\;\text{ effect (\%) = }\left[1-\frac{\left(A_{\text{Sample}}-A_{\text{Sample blank}}\right)}{A_{\text{Control}}}\right]\times 100,$$

where *A*_Control_ was the absorbance of the control (DPPH solution without sample), *A*_Sample_ the absorbance of the test sample (DPPH plus test sample), and the *A*_Sample blank_ the absorbance of the sample only (Sample without DPPH solution). Natural antioxidant, ascorbic acid (AA) was used as positive control.

#### 2.2.4. Reducing Power Assay

Total reducing power was determined as described by Zhu et al. [25] with slight modification. 0.2 mL of the sample solution was mixed with 0.2 mL of phosphate buffer (0.2 M, pH 7.2) and 0.2 mL of 1% potassium ferricyanide. The mixture was incubated at 50°C for 20 min. After incubation, 0.2 mL of trichloroacetic acid (10%) was added. Finally, 0.125 mL of the mixture and 0.125 mL distilled water was dispensed into a 96-well micro plate. To this, 0.02 mL of 0.1% FeCl_3_ was added and absorbance was measured at 655 nm (Bio-Rad, Micro plate reader, Model 680). BHT was used as a positive control for this assay.

#### 2.2.5. Ferrous Ion Chelating Activity

The ferrous ion chelating activity was performed by the method of Decker and Welch [26]. A mixture of sample solution (0.1 mL), distilled water (0.1 mL) and 0.5 mM FeCl_2_ (0.025 mL) was prepared and the absorbance read immediately at 562 nm (Abs 1). Then, 2.5 mM ferrozine (0.025 mL) was added into the mixture and incubated for 20 min at room temperature. The absorbance was measured again (Abs 2). Ethylene diamine tetracetic acid (EDTA) was used as the positive control. The ferrous ion chelating activity was calculated using the following equation;

(2) $$\begin{array}{cc} & \text{Ferrous ion chelating activity [\%]}\\ & \;=\left[1-\frac{\left({\text{Sample}}_{\text{Abs}2}-{\text{Sample}}_{\text{Abs}1}\right)}{\left({\text{Control}}_{\text{Abs}2}-{\text{Control}}_{\text{Abs}1}\right)}\right]\times 100. \end{array}$$

#### 2.2.6. Statistical Analysis

All experiments were conducted in triplicates (*n* = 3) and expressed as mean ± SD. One-way ANOVA test using STATISTICA software (Statsoft, 1999) was utilized to compare the mean values of each treatment and *P*-values < .001 was considered highly significant. The relationships between TPC and DPPH scavenging activity and TPC and metal chelation assay were determined using regression analysis.

## 3. Results

The results illustrate the cytotoxic and antioxidant properties of ME of seven brown seaweeds collected from the Indian coastal waters.

### 3.1. Cytotoxic Activities

The seaweed MEs were evaluated for their cytotoxicity at different concentrations and incubation time exposures and were classified as non-cytotoxic (NCT < 50%), mildly cytotoxic (MCT > 50% but < 75%) and highly cytotoxic (HCT > 75%) based on their lethality to brine shrimp (Table 2).

### 3.2. TPC

FCRs were used to determine TPC of the MEs from *Dictyopteris australis, Dictyopteris delicatula, Padina tetrastromatica, Spatoglossum variabile*, *Spatoglossum aspermum*, *Sargasssum marginatum*, *Stoechospermum marginatum* and the results are shown in Table 3.

### 3.3. DPPH Radical Scavenging Assay

**Figure 1:**
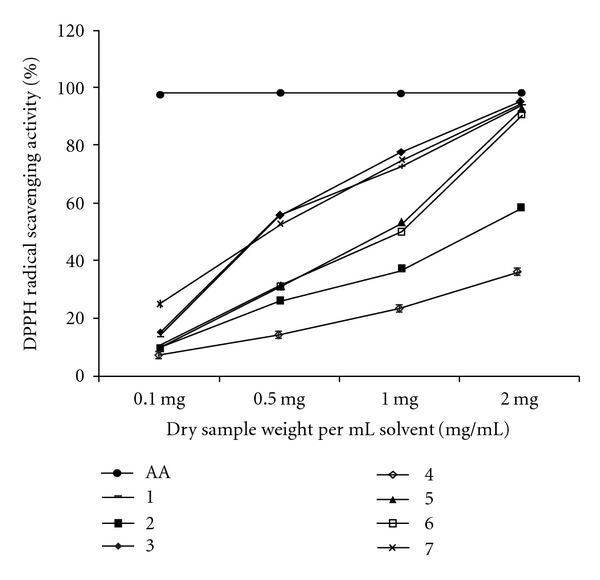
DPPH radical scavenging activity (%) of the total methanolic extracts derived from seven species of brown seaweeds. AA: ascorbic acid. Numbers correspond to the samples in Table 1. Values are presented as means ± SD (*n* = 3).

The radical-scavenging activity of the ME of seven seaweeds assessed were expressed as percentage reduction of the initial DPPH* absorption by the tested compound and is shown in Figure 1. *Stoechospermum marginatum* (IC_50_ 0.56 ± 0.011 mg mL^−1^) displayed significantly (*P* < .001) higher scavenging activity followed by *P. tetrastromatica* (IC_50_ 0.61 ± 0.005 mg mL^−1^)*, D. delicatula* (IC_50_ 0.66 ± 0.002 mg mL^−1^)*, S. aspermum* (IC_50_ 0.98 ± 0.006 mg mL^−1^)*, S. variabile* (IC_50_ 1.01 ± 0.003 mg mL^−1^)*, D. australis* (IC_50_ 1.60 ± 0.013 mg mL^−1^) and *S. marginatum* (IC_50_ 2.87 ± 0.128 mg mL^−1^). Conversely, none of the extracts showed comparable activity to the positive control, AA (IC_50_ 0.07 ± 0.002 mg mL^−1^).

### 3.4. Reducing Power Assay

**Figure 2:**
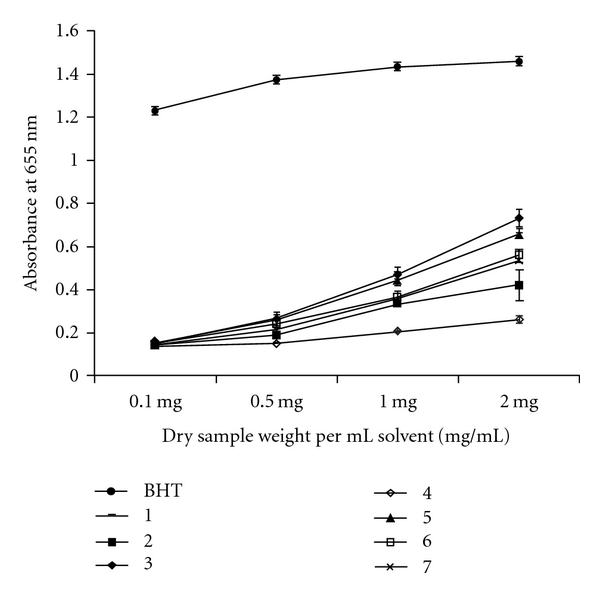
Reducing power of total methanolic extracts derived from seven species of brown seaweeds. BHT: butylated hydroxy toluene. Numbers correspond to the samples in Table 1. Values are presented as means ± SD (*n* = 3).

The MEs of the seven seaweeds were able to reduce Fe^3+^ to Fe^2+^ in a concentration-dependent manner as a function of reducing power. Results obtained showed that the reducing power in ME at all concentrations of 0.1, 0.5 and 1.0 and 2.0 mg mL^−1^ decreased in the following order: BHT *> P. tetrastromatica > D. delicatula > S. aspermum > S. variabile > S. marginatum > D. australis > S. marginatum* (Figure 2). In continuation with the antioxidant activity, the reducing power of MEs also increased with increasing concentration.

### 3.5. Ferrous Ion Chelating Activity

**Figure 3:**
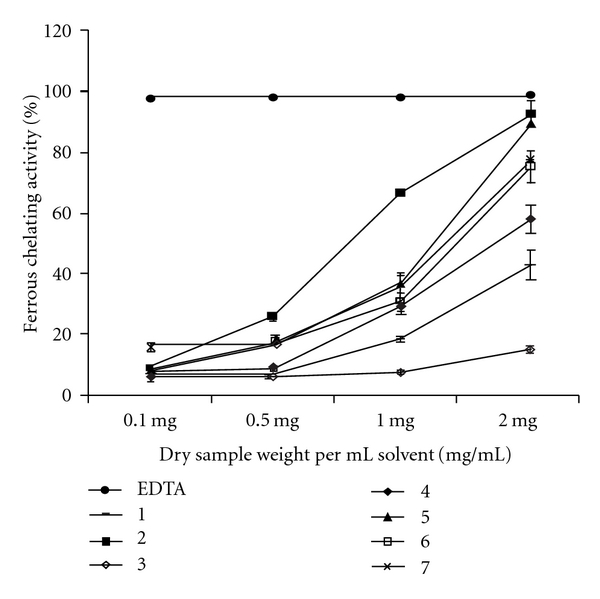
Ferrous ion-chelating activity (%) of total ME from seven species of brown seaweeds. EDTA: ethylene diamine tetra acetic acid. Numbers correspond to the samples in Table 1. Values are presented as means ± SD (*n* = 3).

A reasonably good ferrous ion-chelating efficacy was demonstrated by most of the seaweed extracts in a dose-dependent manner (Figure 3). EDTA (positive control), a strong chelator, demonstrated the best ferrous chelating efficacy (IC_50_ 0.042 ± 0.0008 mg mL^−1^). Amongst all seaweeds, the ferrous chelating efficacy was significantly highest (*P* < .001) for *D. australis* and decreased in the order: *D. australis* (IC_50_ 0.93 ± 0.029 mg mL^−1^) *> S. aspermum (*IC_50_ 1.19 ± 0.020 mg mL^−1^) *> S. marginatum* (IC_50_ 1.30 ± 0.413 mg mL^−1^)*, > S. variabile* (IC_50_ 1.38 ± 0.102 mg m^−1^) *> P. tetrastromatica* (IC_50_ 1.76 ± 0.146 mg mL^−1^) *> D. delicatula* (IC_50_ 2.46 ± 0.247 mg mL^−1^) *> S. marginatum* (IC_50_ 9.17 ± 0.413 mg mL^−1^). 

## 4. Discussions

ROS are molecules or ions formed by the incomplete one-electron reduction of oxygen. They are essentially responsible for the microbicidal activity of phagocytes, regulation of signal transduction and gene expression. Nonetheless, excessive production of ROS by various endogenous and exogenous factors may lead to oxidative stress, loss of cell function and ultimately apoptosis or necrosis. Hence, the balance between production of free radicals and the antioxidant defenses in the body is vital for cell function, regulation and adaptation to diverse growth conditions and has important health implications. Humans have developed a high profile, complex antioxidant patrol including enzymes [such as superoxide dismutases (SOD), catalases (CAT), glutathione peroxidases (GPX)) and small molecule antioxidants (such as ascorbic acid, tocopherol, uric acid and glutathione), forming the first line of defense. The second line of defense against free radical damage is the presence of antioxidants. Polyphenolic antioxidants have been known to play a similar role as endogenous antioxidants and are abundantly found in plants [27, 28]. Seaweed Polyphenols, also called phlorotannins, are vastly different from the terrestrial plants. They are a heterogeneous group of molecules displaying broad range of biological activities and found abundantly in brown seaweeds, forming up to 5–15% of their dried weight [29].

In our studies, we have established that brown seaweeds are a rich source of cytotoxic and antioxidant compounds. Seaweeds, such as *S. aspermum*, *S. marginatum* (cytotoxic studies) and *P. tetrastromatica, S. marginatum* (antioxidant studies), have been investigated earlier; while *D. australis, D. delicatula* and *S. variabile* are first reports in either case.

Brine shrimp assay implies an easy, inexpensive and rapid bioassay for testing cytotoxic activity of plant extracts and can be extrapolated for cell-line toxicity and anti tumor activity. Many scientists have reported cytotoxicity of land plants and algae using brine shrimp as a model organism [30, 31]. Hence, in the present study seven brown seaweeds were screened for cytotoxic activity using the brine shrimp *A. salina* and the activities decreased in the following order; *D. australis* > *S. marginatum* > *Sp. variable* and *Sp. aspermum* > *D. delicatula* > *P. tetrastromatica* > *S. marginatum.* Seaweeds like *D. australis*, *St. marginatum*, *S. variable, S. aspermum* were highly cytotoxic at 100 *μ*g mL^−1^ at 18 and 24 h and caused complete mortality of the brine shrimp at 500 *μ*g mL^−1^ at 24 h duration exposure. A dose-dependent activity was also observed in all seaweeds. In another experiment reported by Ara et al. [31], *S. asperum* was found to be the most cytotoxic to the brine shrimp amongst the seaweeds screened. In our studies, brine shrimp assay of seaweed extracts indicated the existence of potent cytotoxic compounds. This may be sustained by the fact that, several cytotoxic compounds such as fucoidans, laminarins and terpenoids stated to possess anticancer, antitumor and antiproliferative properties are reported to be abundant in seaweeds [4]. These cytotoxic compounds could be further explored as novel leads in cancer chemoprevention and complementary chemotherapy and necessitates further investigation.

Polyphenols are a class of powerful chain-breaking antioxidants with the additional ability to scavenge ROS, inhibit lipid peroxidation as well as chelate metal ions [32–34]. Their radical scavenging ability has been assigned to the number of hydroxyl groups present on them [35]. The TPC of all the seaweeds were expressed as mg gallic acid equivalent (GAE) per gram of seaweed extract (Table 3) ranging from 13.19 ± 0.32 to 25.29 ± 0.445. They varied significantly (*P* < .001) and decreased in the following order: *P. tetrastromatica* > *D. delicatula* > *S. marginatum* > *S. variabile* > *S. aspermum* > *D. australis* > *S. marginatum*. The major active compounds in different seaweed extracts have been reported to be phlorotannins and fucoxanthins [36, 37].

DPPH is a stable radical with a maximum absorbance at 517 nm and is useful for investigating the free radical scavenging activities of various compounds. The method is based on the reduction of alcoholic DPPH solution in presence of a hydrogen donating antioxidant due to formation of a non-radical form of DPPH-H by the reaction and this modification is visually noticeable as a discoloration from purple to yellow [38]. This DPPH radical scavenging ability of the antioxidants has been related to the inhibition of lipid peroxidation. In this study, it was found that all seaweed extracts possessed the ability to scavenge DPPH radical to various degrees in a concentration-dependent manner significantly (*P* < .001). *Sargasssum marginatum* showed the lowest DPPH free radical scavenging activity, while *S. marginatum* had the highest. In addition to *S. marginatum*; *P. tetrastromatica, D. delicatula, S. aspermum* also demonstrated relatively high DPPH radical scavenging activities. The extracts showed superior radical scavenging activity when compared to *Palmaria palmata*, IC_50_ 12.5 mg mL^−1^ [39] and *Kappaphycus alvarezii extracts*, IC_50_ 4.28 mg mL^−1^ [20]. Further analysis revealed that, there was a positive correlation (*R^2^* = 0.396794, *P* < .005) between the TPC and the DPPH radical scavenging activity although not very high; suggesting that not only phenolic constituents, but other components too may have contributed to the scavenging. This may be also explained by the fact that, the properties of polyphenolic compounds vary greatly depending on the number of phenolic groups and hence react differently to the FCR [40].

Reducing capacity is considered as a significant indicator of potential antioxidant activity of a compound or sample [41]. The presence of reductants (i.e., antioxidants) causes the reduction of the Fe^3+^/ferricyanide complex to the ferrous form. Therefore, by measuring the formation of Perl's Prussian blue at 655 nm, the amount of Fe^2+^can be monitored. In the present study, there was a steady increase in reductive potential of all seaweed MEs with increase in concentration (Figure 2). All extracts showed significantly (*P* < .001) higher activities than the negative control but lower activities than the synthetic antioxidant BHT. Extracts could neutralize the free radicals by donating an electron and converting them to a more stable product, ceasing the radical chain reaction in the process to various degrees. Higher absorbance indicated higher reducing power. Similar results were seen in methanol extracts of higher plants as reported by Kumaran and Karunakaran [42]. All concentrations exhibited the OD value <1.0. This was also backed by the findings of Kuda et al. [43].

Ferrozine forms a complex (red color) with Fe^2+^ ion by quantitative interaction. This is, however, disrupted in the presence of chelating agents resulting in a decreased red color formation of the complex. This color reduction when measured gives an estimate of metal chelating ability of the chelator present in the reaction mixture. In this assay, seaweed MEs obstructed the ferrous and ferrozine complex formation implying they have chelating properties. The chelating ability of the seaweed MEs were compared with that of EDTA; a known metal ion chelator. The ferrous ion chelating abilities between the extracts and EDTA are shown in Figure 3. Both the extracts and EDTA showed statistically significant differences (*P* < .001). The highest metal chelating activity was demonstrated by *D. australis* (IC_50_ 0.93 ± 0.029 mg mL^−1^). The metal chelating activity was also concentration dependent. Nevertheless, a very poor correlation of ferrous ion chelating activity with TPC of all seaweeds (*R* ^2^ = 0.077454, *P* > .05) was observed, indicating that phenolic compounds may not be the main chelator of ferrous ions. Metal-binding capacities have been displayed by dietary fibers previously. This is supported by the various reports on the inhibitory effects on ferrous absorption of algal dietary fibers, such as carageenan, agar and alginate [44]. Furthermore, metal ions chelating capacity of phenolic compounds mainly depends on the accessibility of properly oriented functional groups [45] and can no longer bind metals when the phenolic group is conjugated with a carbohydrate group, as in naturally occurring phenolic glycosides [46]. Transition metals, such as iron help superoxide anion (O^*·*−^) (Fenton reaction) and hydrogen peroxide to convert into extremely reactive hydroxyl radical (OH.) (Haber-Weiss reaction) that cause severe damages to membranes, proteins and DNA [47]. They also decompose lipid hydroperoxides into peroxy and alkoxyl radicals and accelerate lipid peroxidation [48]. In the long run, this process can bring about cellular death, carcinogenesis and mutagenesis. An extract with higher iron chelating ability would thus not only inhibit metal dependent oxidative events, but would also be a combatant of ROS-mediated diseases [49].

High intake of antioxidant-rich foods is inversely related to the onset or progression of cancer as revealed by a number of epidemiological studies [50–52]. Indeed, a number of phytochemical antioxidants are known to confer protection against carcinogenic assault, cytotoxic damage to normal cells wrought during cancer therapy and acute and long-term effects of free radicals produced [53, 54]. Nevertheless, further clinical investigations are needed to shed light on the prospective use of antioxidants in prevention and complementary cancer therapy.

**Figure 4:**
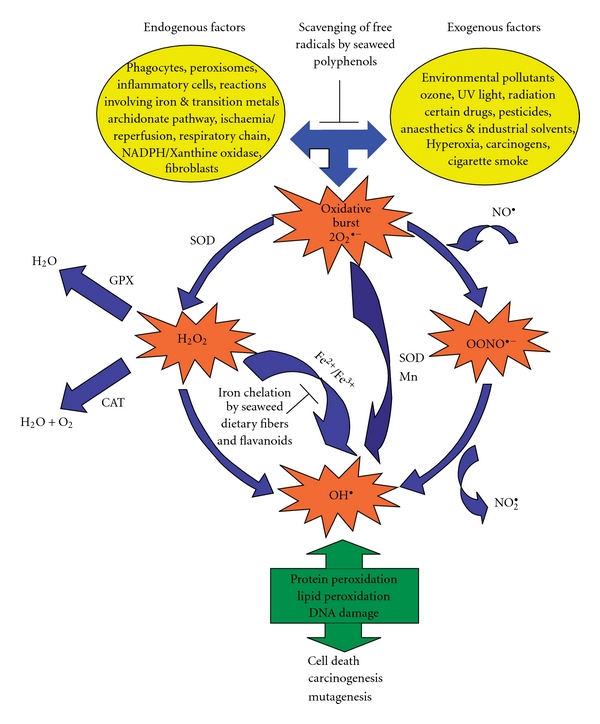
Brown seaweeds and their role in the prevention of ROS-mediated cascade of events. SOD, along with CAT and GPX, forms the first line of the body's antioxidant enzyme defense mechanisms. Various endogenous and exogenous factors give rise to oxidative burst, a phenomenon where superoxide anion radical is the predecessor to majority of ROS and moderator of oxidative chain reactions, which perpetuates the production of secondary ROS. In the long term, this can lead to protein peroxidation, lipid peroxidation and DNA damage within the cell bringing about cell death, carcinogenesis and mutagenesis. The seaweed extracts inhibit these occurrences by preventing the production of ROS at key stages and impeding the inception of cancer and other diseases.

The seaweed MEs investigated in this study have revealed potent cytotoxic and antioxidant activities. The antioxidative constituents possibly play a complimentary role by delaying or preventing the oxidation of cellular oxidizable substrates and selectively inhibiting the ROS cascade of events (Figure 4). All the above data imply a protective role for seaweeds and may prove to be of pharmacological importance, which needs to be explored further.

## Figures and Tables

**Table 1:** List of brown seaweeds evaluated for the experiments.

Sr No	Scientific name	Family	Collected from
Phaeophyta			
(1)	*Dictyopteris australis Lamouroux*	Dictyotaceae	Malvan, Maharashtra
(2)	*Dictyopteryis delicatula Lamouroux*	Dictyotaceae	Anjuna beach, Goa
(3)	*Padina tetrastromatica Hauck*	Dictyotaceae	Baga beach, Goa
(4)	*Sargassum marginatum (C. Agardh) J. Agardh*	Sargassaceae	Marvel beach, Goa
(5)	*Spatoglossum aspermum J. Agardh*	Dictyotaceae	Malvan, Maharashtra
(6)	*Spatoglossum variabile Figari & De Notaris*	Dictyotaceae	Anjuna beach, Goa
(7)	*Stoechospermum marginatum (C. Agardh) Kutzing*	Dictyotaceae	Marvel beach, Goa

**Table 2:** Cytotoxicity activity of seven brown seaweed extracts using brine shrimp lethality assay.

Sample	Concentration (*μ*g)	6 h	Cytotoxicity	18 h	Cytotoxicity	24 h	Cytotoxicity
(K_2_Cr_2_O_7_)	50	1.66 ± 2.58	NCT	68.33 ± 6.83	MCT	100.00 ± 0.00	HCT
	100	10.00 ± 4.47	NCT	68.33 ± 2.58	MCT	100.00 ± 0.00	HCT
	500	100.00 ± 0.00	HCT	100.00 ± 0.00	HCT	100.00 ± 0.00	HCT

*Dictyopteryis australis*	50	41.66 ± 6.83	NCT	100.00 ± 0.00	HCT	100.00 ± 0.00	HCT
*Dictyopteryis delicatula*	50	25.00 ± 4.47	NCT	26.66 ± 2.58	NCT	41.66 ± 2.58	NCT
*Padina tetrastromatica*	50	11.66 ± 2.58	NCT	20.00 ± 0.00	NCT	25.00 ± 4.47	NCT
*Sargassum marginatum*	50	10.00 ± 0.00	NCT	11.66 ± 2.58	NCT	15.00 ± 0.00	NCT
*Spatoglossum aspermum*	50	0	NCT	43.33 ± 6.83	NCT	58.33 ± 9.31	MCT
*Spatoglossum variable*	50	1.66 ± 2.58	NCT	68.33 ± 2.58	MCT	71.66 ± 13.66	MCT
*Stoechospermum marginatum*	50	8.33 ± 2.58	NCT	73.33 ± 2.58	MCT	86.66 ± 5.16	HCT

*Dictyopteryis australis*	100	53.33 ± 5.16	MCT	100.00 ± 0.00	HCT	100.00 ± 0.00	HCT
*Dictyopteryis delicatula*	100	28.33 ± 6.83	NCT	30.00 ± 4.47	NCT	51.66 ± 2.58	MCT
*Padina tetrastromatica*	100	20.00 ± 0.00	NCT	25.00 ± 4.47	NCT	36.66 ± 2.58	NCT
*Sargassum marginatum*	100	23.33 ± 2.58	NCT	23.33 ± 2.58	NCT	23.33 ± 2.58	NCT
*Spatoglossum aspermum*	100	6.66 ± 2.58	NCT	86.66 ± 2.58	HCT	93.33 ± 2.58	HCT
*Spatoglossum variable*	100	33.33 ± 5.16	NCT	91.66 ± 2.58	HCT	93.33 ± 2.58	HCT
*Stoechospermum marginatum*	100	16.66 ± 2.58	NCT	90.00 ± 4.47	HCT	91.66 ± 9.31	HCT

*Dictyopteryis australis*	500	90.00 ± 4.47	HCT	100.00 ± 0.00	HCT	100.00 ± 0.00	HCT
*Dictyopteryis delicatula*	500	30.00 ± 4.47	NCT	31.66 ± 2.58	NCT	56.66 ± 6.83	MCT
*Padina tetrastromatica*	500	23.33 ± 2.58	NCT	31.66 ± 5.16	NCT	41.66 ± 6.83	NCT
*Sargassum marginatum*	500	25 ± 7.75	NCT	31.66 ± 2.58	NCT	36.66 ± 6.83	NCT
*Spatoglossum aspermum*	500	30.00 ± 0.00	NCT	96.66 ± 5.16	HCT	100.00 ± 0.00	HCT
*Spatoglossum variable*	500	100.00 ± 0.00	HCT	100.00 ± 0.00	HCT	100.00 ± 0.00	HCT
*Stoechospermum marginatum*	500	60.00 ± 8.94	MCT	100.00 ± 0.00	HCT	100.00 ± 0.00	HCT

NCT: non-cytotoxic; MCT: mildly cytotoxic; HCT: highly cytotoxic.

**Table 3:** TPC of seven brown seaweeds expressed as GAE; mg g^−1^ of methanol extract (*n* = 3).

Seaweed species	GAE; mg g^−1^ of total methanolic extract
Phaeophyceae	
*Dictyopteris australis*	13.37 ± 0.140
*Dictyopteryis delicatula*	21.34 ± 0.428
*Padina tetrastromatica*	25.29 ± 0.445
*Sargassum marginatum*	13.19 ± 0.32
*Spatoglossum aspermum*	14.13 ± 0.046
*Spatoglossum variable*	14.85 ± 0.093
*Stoechospermum marginatum*	20.04 ± 0.382

All the values are mean ± SD; SD: standard deviation significant at *P* < .001.
